# The role of enhanced expression of Cx43 in patients with ulcerative colitis

**DOI:** 10.1515/med-2023-0885

**Published:** 2024-05-18

**Authors:** Weidong Liu, Yan Feng, Ting Li, Tian Shi, Wenjia Hui, Huan Liu, Feng Gao

**Affiliations:** Department of Gastroenterology, People’s Hospital of Xinjiang Uygur Autonomous Region, Urumqi, 830000, China; Xinjiang Clinical Research Center for Digestive Diseases, Urumqi, 830000, China

**Keywords:** connexin 43, gap junction, ulcerative colitis, mucosal immunity

## Abstract

The pathogenesis of ulcerative colitis (UC) involves chronic inflammation of the submucosal layer and disruption of epithelial barrier function within the gastrointestinal tract. Connexin 43 (Cx43) has been implicated in the pathogenesis of intestinal inflammation and its associated carcinogenic effects. However, a comprehensive analysis of Cx43’s role in mucosal and peripheral immunity in patients with UC is lacking. In this study, the colon tissues of patients with UC exhibited severe damage to the intestinal mucosal barrier, resulting in a significant impairment of junctional communication as observed by transmission electron microscopy. The mRNA expression of Cx43 was found to be significantly elevated in the UC group compared to the control group, as determined using the Affymetrix expression profile chip and subsequently validated using qRT-PCR. The immunofluorescence analysis revealed a significantly higher mean fluorescence intensity of Cx43 in the UC group compared to the control group. Additionally, Cx43 was observed in both the cell membrane and nucleus, providing clear evidence of nuclear translocation. The proportion of Cx43 in the UC group for CD4^+^ and CD8^+^ T lymphocytes was increased in the control group, but only the proportion of Cx43 for CD8^+^ T lymphocytes showed significant difference by flow cytometry. The involvement of Cx43 in the pathogenesis of UC and its potential role in mucosal immunity warrants further investigation, as it holds promise as a prospective biomarker and therapeutic target for this condition. The proportion of Cx43 in the UC group for CD4^+^ and CD8^+^ T lymphocytes was increased in the control group, but only the proportion of Cx43 for CD8^+^ T lymphocytes showed a significant difference.

## 1 Introduction

Ulcerative colitis (UC) is a chronic, non-specific inflammatory bowel disease characterized by recurrent episodes, prolonged symptoms, diverse extra-intestinal manifestations, and an increased risk of malignancy, significantly impacting the patients’ quality of life [1]. UC is highly associated with a risk of colon cancer and has two important biological characteristics: defective intestinal mucosal barrier and dysregulation of innate immunity. Intestinal mucosal barrier defect is one of the important pathogenesis factors of UC, while gap junction is a crucial structure in the intestinal mucosal barrier. Under inflammatory conditions, the expression change and redistribution of the junction are closely related to intestinal immune dysfunction and intestinal mucosal barrier defects [2,3]. The expression of connexin is tissue-specific, as for colonic tissues, which are Cx26, Cx31, Cx31.1, Cx32, Cx36, Cx40, Cx43, and Cx45 [4,5]. The aim of this study was to examine the differences in the connexin 43 (Cx43) expression in colon tissues of patients with UC to determine whether Cx43 plays a role in the progression of UC.

## 2 Materials and methods

### 2.1 Patients and public involvement

During the colonoscopy procedure, the active involvement of patients and the general public was incorporated into the study design. Patients have received oral and written information about this trial; otherwise, they has not been involved in the recruitment and conduct of the study. The diagnostic criteria of UC were based on Chinese consensus on the diagnosis and treatment of inflammatory bowel disease (Beijing, 2018) [6]. Experimental specimens were obtained from patients diagnosed with UC in the Gastroenterology Department of Xinjiang Uygur Autonomous Region People’s Hospital from March 2019 to July 2021. In this study, nine patients with UC and nine healthy control patients were recruited. Twenty-four colon tissue samples were obtained for transmission electron microscopy, immunofluorescence, and qPCR studies, respectively, and six blood samples were used for flow cytometry. All patients signed an informed consent.

### 2.2 Transmission electron microscopy

Colon specimens were collected from three UC patients and the control group (one case in each group). The biopsy specimens were fixed in 2.5% glutaraldehyde immediately and kept overnight at 4℃. The next day, the fixed specimens were taken out and washed with PBS, fixed with osmic acid, dehydrated with ethanol series, soaked with epoxy resin overnight, and fixed in the mold. Afterward, these fixed tissue repair blocks were sliced with an ultrathin slicer, and the samples were stained with sodium acetate and lead citrate for 20 min, which were finally observed under a transmission electron microscope.

### 2.3 Affymetrix mRNA expression spectrum chip detection

Colonic tissue samples of control and UC groups, four samples for each group, were obtained from colonoscopy biopsy, which were stored in liquid nitrogen. Affymetrix mRNA expression spectrum chip was used to detect the total RNA of the sample as the starting point, and it was performed by Beijing Boao Jindian Biotechnology Co., Ltd. Amplification and biotin labeling were performed *in vitro*. The labeling process was performed using the Ambion #1792 cRNA amplification marker kit.

### 2.4 Immunofluorescence analysis

One colon specimen was taken from each of the three UC patients and the control group. The experimental specimens were fast-frozen in phosphate buffer containing 4% paraformaldehyde within 48 h. After removal of endogenous peroxidase 3% hydrogen peroxide was used and Cx43-specific antibodies were added and incubated overnight at 4℃. The next day, FITC was added to mark the two resistances, and the Cx43 expression level and position distribution were detected using a fluorescence microscope, which was kept away from light according to the instructions of operation.

### 2.5 Real-time quantitative PCR

Two colon specimens were collected from each of the three UC patients and the control group. The total RNA was extracted by the Trizol one-step method, and dissolved in 11 μl of DEPC water (1‰). Total RNA (3 μl) was synthesized with oligo(dT) Perimer as a primer. About 3 μl of reverse transcriptase was used as a template, followed by substrate, target primer, internal reference primer, and sterile double distilled water (Table 1). PCR synthesis was performed. All cDNA samples were configured with real-time quantitative PCR reaction system and placed on a real-time quantitative PCR instrument for the amplification reaction. The amplification primers of Cx43 and β-actin were predenatured at 95℃ for 30 s, followed by a total of 40 cycles at 95℃ for 30 s, 60℃ for 30 s, and finally extended at 72℃ for 5 min. In this study, β-actin was used as an internal reference. The *C*_t_ values of the target mRNA and β-actin for each sample were determined using the same computer, following the aforementioned steps. Subsequently, the relative expression level of Cx43 mRNA was calculated by evaluating 2^−△△^Ct for each individual sample.

**Table 1:** Primer sequence

Gene	Primer sequence
Cx43	F: CAA TCT CTC ATG TGC GCT TCT
	R: GGC AAC CTT GAG TTC CTC T
β-actin	F: CAT GTA CGT TGC TAT CCA GGC
	R: CTC CTT AAT GTC ACG CAC GAT

### 2.6 Flow cytometry

Whole blood samples were collected from three UC patients and three healthy volunteers. The operation was carried out according to the instructions of the peripheral blood lymphocyte separation kit. The isolated lymphocyte suspension was subpacked in centrifugal tubes, the number of cells was controlled at about 500,000 per tube, and 50 μl of blocking serum was added at 4℃ for 30 min. The cells were washed with 1 ml of PBS, centrifuged at 2,400 rpm at 4℃ for 5 min, and then the supernatant was discarded. According to the recommended dose, to dilute the primary antibody in sterile PBS, 50 μl of PBS containing the primary antibody was added, mixed, and incubated at 4℃ for 30 min. The cells were washed under the same conditions and 50 μl of PBS containing secondary antibodies was added, mixed, and incubated at 4℃ for 30 min. The cells were washed under the same conditions, resuspended the cells with 200 μl of PBS followed by analysis on a flow cytometer.

### 2.7 Statistical analysis

Statistical analysis was performed using SPSS 17.0 software, and numerical variables were described in terms of mean ± SD. Two independent samples *t*-tests were used to compare the differences between the two groups, one-way ANOVA was used for the comparison between the groups, the LSD method was used for the multiple comparisons between the groups, and *P* < 0.05 was considered statistically significant.


**Ethical approval**: The study was approved by the Ethics Committee of People’s Hospital of Xinjiang Uygur Autonomous Region. All patients provided written informed consent.

## 3 Results

### 3.1 Ultrastructure of intestinal epithelial cells

**Figure 1:**
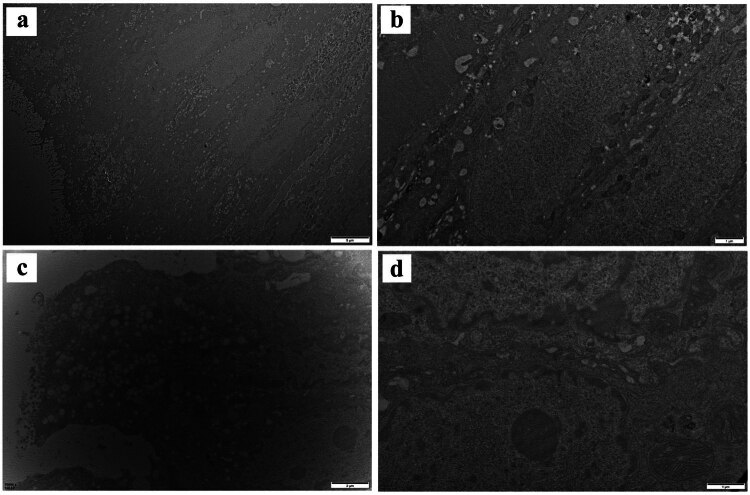
Ultrastructure of intestinal epithelial cells in normal and UC patients: (a) normal control intestinal epithelial cells; (b) connection structure between intestinal epithelial cells; (c) UC intestinal epithelial cells; and (d) intercellular connection structure of UC.

Transmission electron microscopy revealed that the intestinal epithelial cells in the normal control group exhibited a well-organized structure and abundant microvilli, with complete and tight cell junctions. However, in patients with UC, the intestinal mucosal barrier was severely damaged, villi of the intestinal mucosal epithelium were destroyed, the cell gap was significantly widened, cell vacuolation was serious, and the gap junction communication structure between cells was significantly weakened (Figure 1).

### 3.2 Expression of Cx43 mRNA

**Figure 2:**
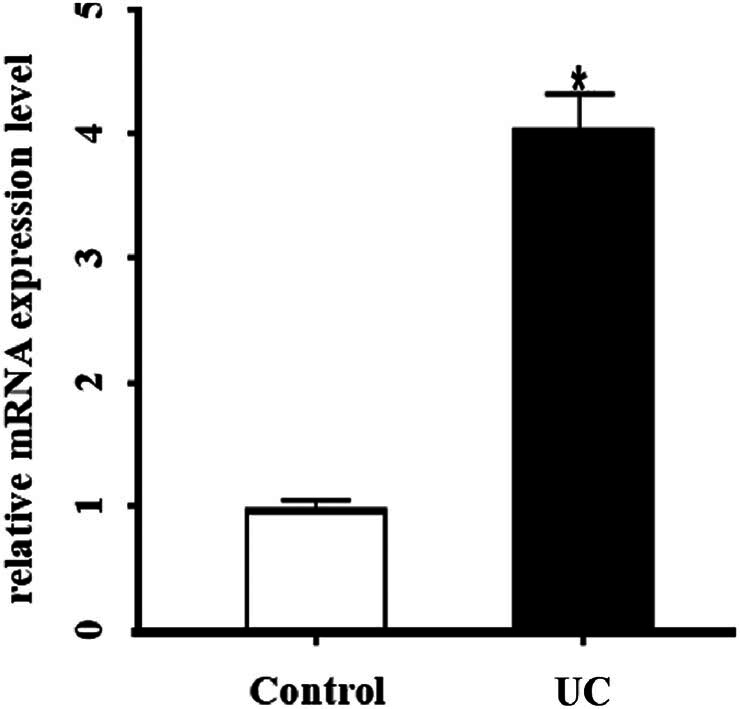
Relative expression levels of Cx43 in control and UC patients analyzed by qRT-PCR.

The mRNA expression differences between the UC group and control group were analyzed using the Affymetrix expression spectrum chip (Appendix). The results demonstrated a decrease in the expression levels of Cx26 and Cx32, while there was a significant increase in the expression levels of Cx43 and Cx45 in the UC group (Table 2). The expression of Cx43 mRNA was validated using real-time quantitative PCR, revealing a significantly higher relative expression level in the UC group compared to the control group (*P* < 0.01; see Figure 2).

**Table 2:** Connexin expression in control and UC patients using the Affymetrix mRNA expression spectrum chip

Name	Gene symbol	Chromosomal location	Score (*d*)	*q*-value (%)	Fold change
Cx26	GJB2	chr13q11-q12	−1.68	3.74	0.83
Cx32	GJB1	chrXq13.1	−2.44	1.28	0.7
Cx43	GJA1	chr6q22.31	3.78	0.35	1.75
Cx45	GJC1	chr17q21.31	9.92	0	2.0

### 3.3 Expression and distribution of Cx43

**Figure 3:**
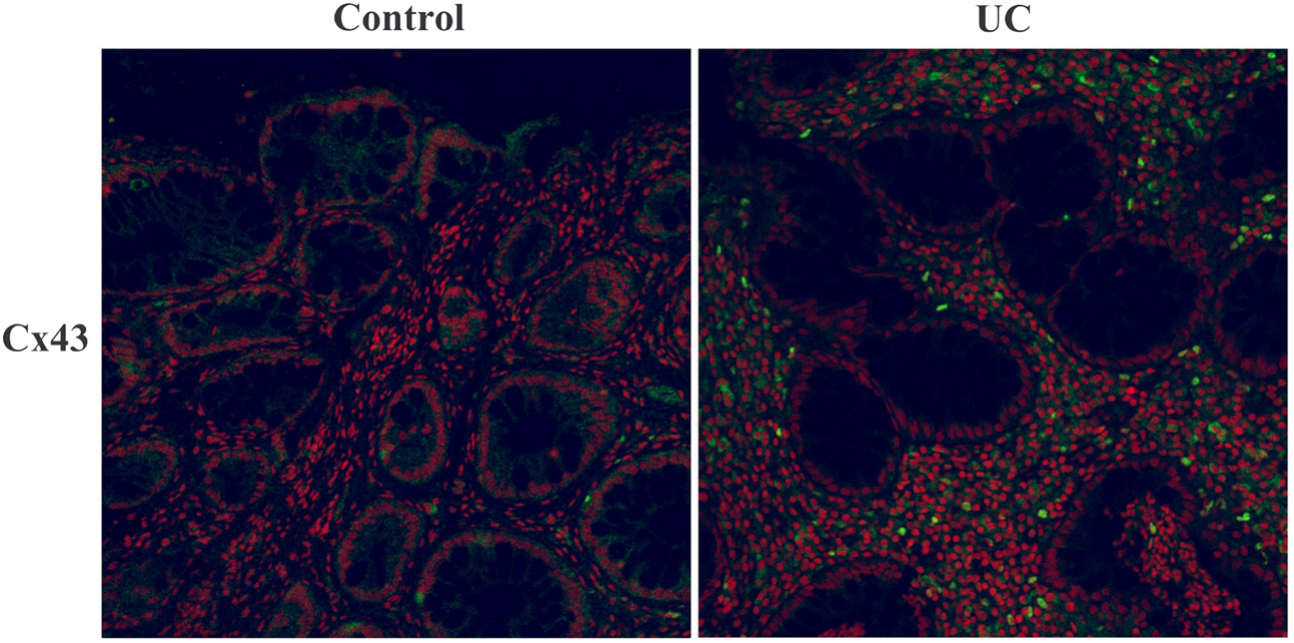
Immunofluorescence analysis of Cx43 expression and distribution (400×).

The nucleus is indicated by red labeling, while Cx43 is labeled in green. The mean fluorescence density of Cx43 in the UC group was significantly higher compared to the control group (65.1 ± 3.7 vs 51.3 ± 3.3, *P* < 0.01). Fluorescence labeling of Cx43 was observed in both the cytoplasm and cell membrane, while in the UC group, there was evident translocation of Cx43 into the nucleus (Figure 3).

### 3.4 Proportion of Cx43 in CD4^+^ and CD8^+^ T lymphocytes

**Figure 4:**
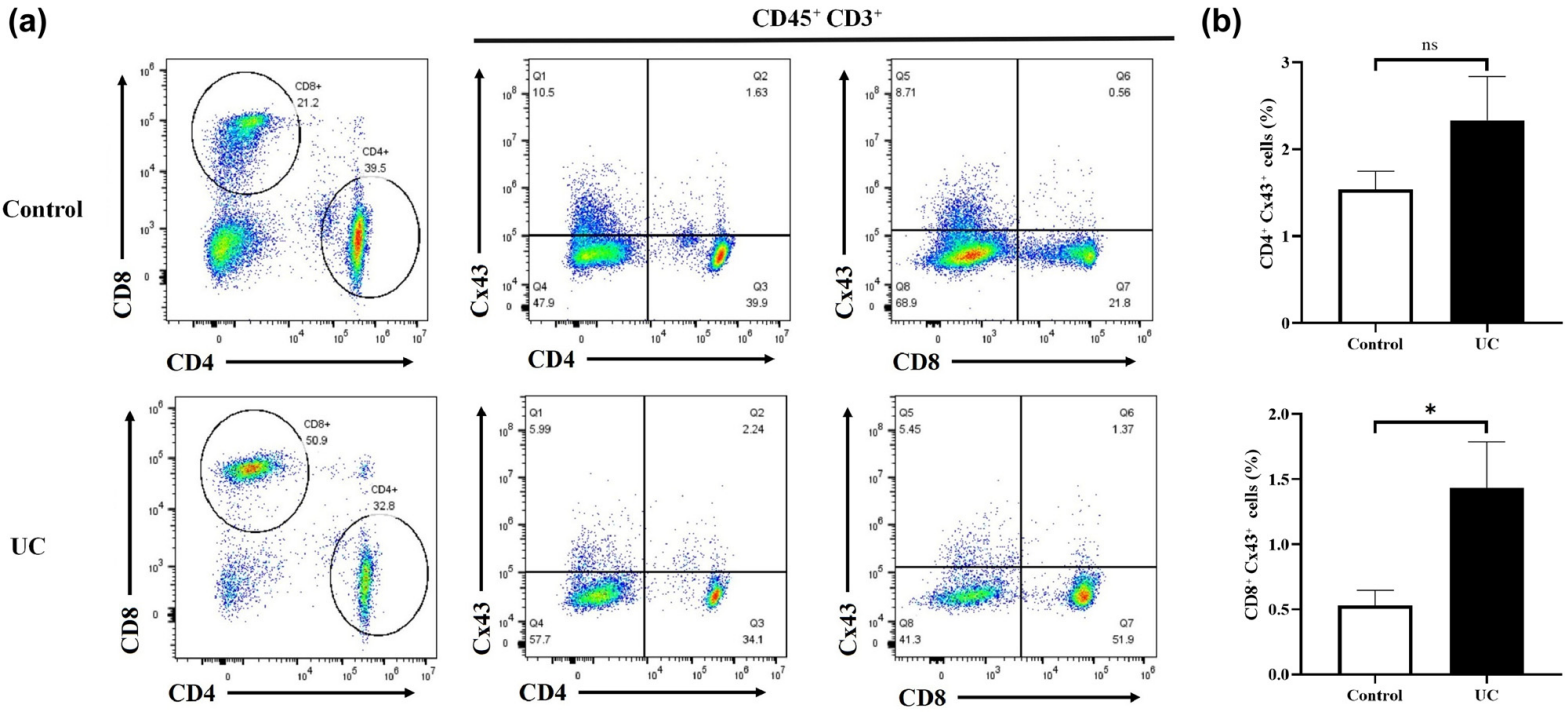
The proportion of Cx43 in CD4^+^ and CD8^+^ T lymphocytes.

The proportion of Cx43 in CD4^+^ and CD8^+^ T lymphocytes was detected using a flow cytometer. The proportion of Cx43 in the UC group (1.8 ± 0.72%) for CD4^+^ T lymphocytes had no difference with the control group (1.6 ± 0.16%; *P* > 0.05). A significantly increased proportion of Cx43 was observed in the UC group (1.4 ± 0.35%) for CD4^+^ T lymphocytes compared with that in the control group (0.53 ± 0.12%; *P* < 0.05) (Figure 4).

## 4 Discussion

UC is a chronic, non-specific inflammatory bowel disease characterized by recurrent episodes, prolonged symptoms, diverse extra-intestinal manifestations, and an increased risk of malignancy, significantly impacting the patients’ quality of life [1]. Currently, UC has emerged as a prevalent gastrointestinal disorder and the primary etiology of chronic diarrhea. The pathogenesis is associated with defects in the intestinal mucosal barrier and immune dysfunction within genetically susceptible individuals, influenced by various factors such as persistent intestinal infections and environmental changes [7]. Gap junctions as a conduit for direct intercellular communication are closely associated with the impairment of intestinal barrier function and immune dysfunction, both of which play pivotal roles in the pathogenesis of UC. Furthermore, the intestinal mucosal barrier serves as a crucial determinant during UC development, while also functioning as an essential organ involved in stress response regulation and production of inflammatory mediators. Gap junction protein expression levels vary in various inflammatory diseases and different stages of the tumor process and can interact with proteins associated with the cell cycle or tumor formation and participate in immune regulation *in vivo* [8]. However, the intestinal mucosal tissue of UC exhibited severe damage and a widening of the intercellular space, potentially leading to alterations in the gap junction function.

The gap junction is a special membrane structure formed by joining the connection channels between two adjacent cells, of which the basic unit is connexin (Cx). In humans, more than 20 connexin isoforms have been identified. On the cellular membrane, six connexins assemble to form a hemichannel, and two hemichannels from adjacent cells dock together via hydrogen bonding to establish a compatible and functional gap junction channel [9]. The gap junction channel allows passing molecules with a relative molecular mass of less than 1 kDa, including ATP, cAMP, IP_3_, and ions. Thus, through gap junctions, adjacent cells can exchange information, energy, and substances, participate in the metabolic coupling of the intercell material exchange and electrical coupling of electrical signal transmission, and play a crucial regulatory role in physiological processes such as cell metabolism, homeostasis, proliferation, and differentiation [10]. Using the Affymetrix expression profile chip analysis, this study found that, in patients with UC, the expression levels of Cx43 and Cx45 were significantly increased, while Cx26 and Cx32 decreased. In patients with UC, the results indicated that gap junctions also play an important role in the development of colorectal tumors.

The expression of Cx43 is abundant in human intestinal epithelial cells and mucosal muscularis. Studies have revealed a significant decrease in the gap junction protein expression along with cytoplasmic membrane relocation during the progression of colorectal cancer [11,12], Cx43 in the samples of colon cancer was significantly lowered by 75% than that in the control group, with the characteristics of the tumor suppressor, and organization and tumor invasion was significantly associated with cancer [13]. In addition, previous studies have also shown that Cx43 expression may inhibit the growth of colon cancer cells by interfering with the Wnt/β-catenin pathway [14]. The Wnt/β-catenin pathway plays a role in the formation of epithelial homeostasis in intestinal physiology and is the central organizer of intestinal epithelial stem cell recognition and maintenance. It is not only important for regulating embryonic growth but also for intestinal self-renewal. The overactivated signaling pathway is a key factor in the development of UC-related colorectal cancer [15,16].

However, there is a paucity of studies investigating the role of gap junction proteins in patients with UC. Immunofluorescence analysis reveals an upregulation of Cx43 protein expression and its translocation to the nucleus. Recent research has elucidated a crosstalk between the Wnt signaling pathway and Cx43, whereby activation of Wnt signaling governs the nuclear translocation of Cx43 in cellular systems [12]. The expression of Cx26 and Cx43 was observed to be upregulated and redistributed in the basement membrane under inflammatory conditions, thereby facilitating the formation of functional gap junction channels between intestinal epithelial cells and macrophages [17]. However, a separate study revealed that mouse macrophages exert regulatory control over the functionality of intestinal epithelial cells via gap junctions [18]. Heteromorphic gap junction communication and paracrine between immune cells and intestinal epithelial cells are crucial factors in regulating the function of epithelial cells, establishing connective complexes between inflammatory cells and epithelial cells, and promoting the functional deficiency of the intestinal epithelial mucosal barrier [19]. The pathogenesis of UC involves chronic inflammation of the submucosal layer and disruption of epithelial barrier function within the gastrointestinal tract. Achieving complete restoration of the mucosal integrity represents a primary therapeutic objective for long-term remission in UC. Functionally, Wnt activation can increase the rate of wound reepithelization in rat skin *in vivo*. Recent studies have shown that the Wnt signaling pathway can promote mucosal repair in TNBS-treated mice [20].

The pathogenesis of UC primarily arises from dysregulation of mucosal immunity, genetic factors, and environmental influences. A cascade of intricate and pivotal events ensues within the immune system. Some immune cells migrate to the infected site, and the normal process of lymphocyte homing determines the function of the intestinal mucosal immune barrier. Cx43 is a main gap junction protein expressed in immune cells [21]. Studies have demonstrated that Cx43 may not participate in the normal development of the immune system but may play a role in antigen cross-presentation, dendritic cell maturation, T cell development, and regulatory T cell function. Intercellular communication mediated by gap junctions between DCs is necessary for the effective activation of DCs, and the knockdown of Cx43 in bone marrow-derived DCs by small interfering RNA can lead to diminished T-cell stimulation [22,23,24]. As an important indicator of immune system function, T lymphocyte subsets CD4^+^ T cells and CD8^+^ T cells play a major role. CD4^+^ T lymphocytes and helper T cells assist humoral and cellular immunity, and CD8^+^ T lymphocytes and cytotoxic T cells kill target cells and are activated by direct binding of MHC I with antigen [25]. During the immune response, many immune cells undergo maturation and become functionally enhanced cells, enabling them to respond to specific types of pathogens in specific ways [18]. Our results show that the expression of Cx43 in peripheral blood T cells is upregulated in individuals with UC, potentially contributing to the mucosal inflammatory response associated with UC.

Cx43, as the most widely distributed and abundant protein in the gap junction family, plays a pivotal role in mediating information, energy, and substance exchange between cells. The expression of Cx43 is intricately linked to both local and systemic immune responses [26]. Functionally, Cx43 forms gap junction channels that actively participate in various physiological or pathological processes within tissues and organs. Additionally, non-classical functions of Cx43 hexamers have been identified in structures such as the plasma membrane, mitochondrial intima, and extracellular vesicle surface, contributing to the promotion of inflammatory disease mechanisms [27]. Leveraging these dual functionalities presents an opportunity for targeted therapeutic drug development and biomarker identification for related diseases.

In summary, the gap junction serves as a crucial means of intercellular communication, and connexins hold promise as potential prognostic markers and therapeutic targets for UC. By analyzing the expression of connexin in patients with UC, we have discovered that Cx43 actively participates in the inflammatory process. Specifically, the nuclear translocation of Cx43 may play a pivotal role in the pathogenesis of UC. The mechanism of gap junction proteins involved in UC needs further investigation.

## Figures and Tables

**Figure S1:**
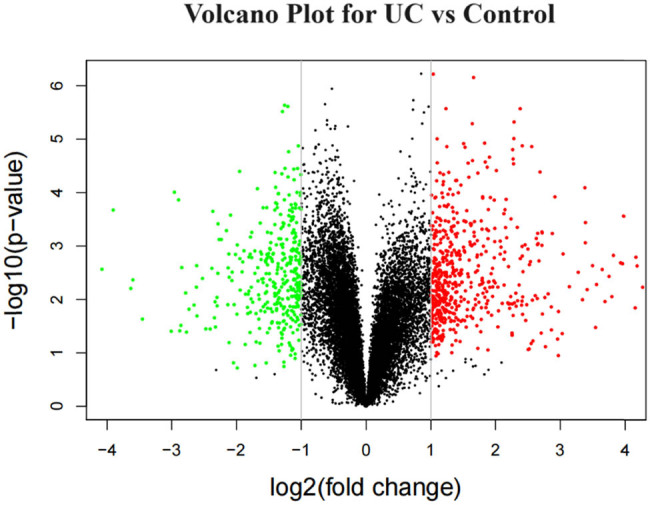
Volcano plot for UC vs Control.

**Figure S2:**
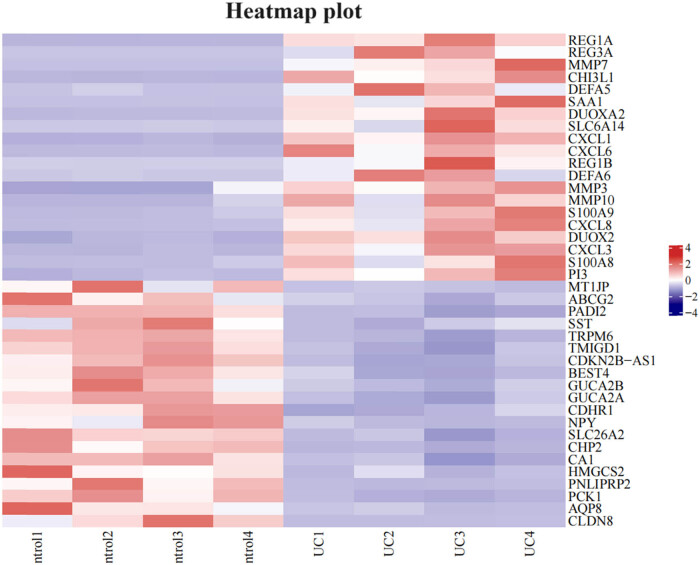
Heatmap plot of differential genes for UC vs Control.
